# Towards an optimal therapy strategy for myogenous TMD, physiotherapy compared with occlusal splint therapy in an RCT with therapy-and-patient-specific treatment durations

**DOI:** 10.1186/s12891-017-1404-9

**Published:** 2017-02-10

**Authors:** Robert J. van Grootel, Rob Buchner, Daniël Wismeijer, Hilbert W. van der Glas

**Affiliations:** 1 Meander Medical Centre, Amersfoort, The Netherlands; 2 Sleen, The Netherlands; 3 Department of Oral Implantology and Prosthetic Dentistry, Academic Centre for Dentistry Amsterdam (ACTA), VU University and University of Amsterdam, Amsterdam, The Netherlands; 4 Department of Otorhinolaryngology and Head & Neck Surgery, University Medical Centre Utrecht, Utrecht, The Netherlands; 5 The Dental School, University of Dundee, Park Place, Dundee, DD1 4HR Scotland UK

**Keywords:** Temporomandibular disorders, Myofascial pain syndrome, Randomized controlled trial, Stepped-care, Medical decision, Physiotherapy, Occlusal splint

## Abstract

**Background:**

Temporomandibular Disorders (TMD) may be characterized by pain and restricted jaw movements. In the absence of somatic factors in the temporomandibular joint, mainly myogenous, psychobiological, and psychosocial factors may be involved in the aetiology of myogenous TMD. An occlusal appliance (splint) is commonly used as a basic therapy of the dental practice. Alternatively, a type of physiotherapy which includes, apart from massage of sore muscles, aspects of cognitive-behavioural therapy might be a basic therapy for myogenous TMD. Treatment outcome of physiotherapy (Ph-Tx) was evaluated in comparison to that of splint therapy (Sp-Tx), using the index Treatment Duration Control (TDC) that enabled a randomized controlled trial with, comparable to clinical care, therapy-and-patient-specific treatment durations.

**Methods:**

Seventy-two patients were randomly assigned to either Ph-Tx or Sp-Tx, with an intended treatment duration between 10 and 21 or 12 and 30 weeks respectively. Using TDC, the clinician controlled treatment duration and the number of visits needed. A blinded assessor recorded anamnestic and clinical data to determine TDC-values following treatment and a 1-year follow-up, yielding success rate (SR) and effectiveness (mean TDC) as treatment outcomes. Cohen’s *d*, was determined for pain intensity. Overall SR for stepped-care was assessed in a theoretical model, *i.e*. a second of the two studied therapies was applied if the first treatment was unsuccessful, and the effect of therapy sequence and difference in success rates was examined.

**Results:**

SR and effectiveness were similar for Ph-Tx and Sp-Tx (long-term SR: 51–60%; TDC: −0.512– −0.575). Cohen’s *d* was 0.86 (Ph-Tx) and 1.39 (Sp-Tx). Treatment duration was shorter for Ph-Tx (on average 10.4 weeks less; *p* < 0.001). Sp-Tx needed 7.1 less visits (*p* < 0.001).

**Conclusions:**

Physiotherapy may be preferred as initial therapy over occlusal splint therapy in stepped-care of myogenous TMD. With a similar SR and effectiveness, physiotherapy has a shorter duration. Thus patients whose initial physiotherapy is unsuccessful can continue earlier with subsequent treatment. The stepped-care model reinforces the conclusion on therapy preference as the overall SR hardly depends on therapy sequence.

**Trial registration:**

isrctn.com/ISRCTN17469828. Retrospectively registered: 11/11/2016

**Electronic supplementary material:**

The online version of this article (doi:10.1186/s12891-017-1404-9) contains supplementary material, which is available to authorized users.

## Background

Temporomandibular Disorders (TMD) is a collective term embracing a number of clinical problems including conditions of the masticatory musculature, the temporomandibular joint and associated structures, or both [1]. These disorders are characterized by pain and restricted jaw movements. Inter-therapy outcome was studied for a basic type of TMD. To that end, patients were selected who have muscle disorders alone, corresponding to the myofascial subtype according to the Research Diagnostic Criteria, RDC/TMD [2], further denoted as myogenous TMD.

In the absence of somatic factors in the temporomandibular joint, mainly myogenous, psychobiological, and psychosocial factors may be involved in the aetiology of myogenous TMD [3, 4]. Regarding myogenous factors, external events, for example, trauma, may initiate peripheral sensitization of nociceptors in the masticatory muscles [5]. Psychobiological factors include those related to central sensitization and dysfunctional descending pain modulation [3, 5, 6]. Psychosocial factors related to stress, emotion and somatic awareness interact with psychobiological factors [3, 4].

Therapy with an occlusal appliance (splint) is commonly used as a basic TMD treatment in the dental practice [7]. An occlusal splint will alter afferent activity from intra-oral tissues by covering teeth and providing pressure on intra-oral tissues. Furthermore, an occlusal splint alters the position of the temporomandibular joint by increasing the vertical dimension of the mouth. In motor control, a splint changes activity patterns of the jaw closing muscles during clenching [8, 9]. As splint therapy is not specific, the mechanisms underlying its effectiveness are not well understood [10, 11]. In view of aetiology, a type of physiotherapy which includes, apart from massage of sore muscles, aspects of cognitive-behavioural therapy, might be a basic therapy for myogenous TMD rather than splint therapy. Regarding other features, the extent of a patient’s active participation is larger for physiotherapy. Furthermore, patients whose physiotherapy is ended successfully have learned techniques to avoid stress and to relieve pain, which can be used if they feel a need afterwards. Such techniques may avoid the need of using an intra-oral appliance in the long-term. It is therefore of interest to compare treatment outcome of physiotherapy to that of splint therapy.

The effectiveness of occlusal splint therapy has previously been compared with other therapies or conditions using traditional Randomized Controlled Trials (RCTs), with a constant period of treatment of 6 weeks to 3 months [12–18]. In clinical care, however, the duration of treatment varies as it depends on the type of therapy as well as on a patient’s speed of recovery. When therapies of TMD differ in mean duration, a constant period of evaluation might influence an assessment of success rate and effectiveness. A short period will favour short therapies whereas a long period might be disadvantageous by including post-treatment changes in the outcome.

For example, when a therapy with a mean duration of 2 months, is arbitrarily ended after 1 month for evaluation, it will be unsuccessful in those patients who respond so slowly that a residual level of signs and symptoms of TMD is not reached after 1 month. Part of these patients will attain a residual level with a longer treatment. Hence, the percentage of successful treatments across the entire patient sample of a therapy (success rate) will be biased towards a smaller value when therapy evaluation is carried out before treatments have been completed. An early evaluation also yields a smaller improvement in an outcome variable, for example pain intensity, of slowly responding patients. Hence, therapy effectiveness which is related to the degree of improvement averaged across the entire patient sample, will then be biased towards a smaller value. When two therapies, *A* and *B*, are both arbitrarily evaluated after a relatively long period, for example 3 months, while the mean duration of these therapies is 1 and 2 months respectively, the therapy outcome might be confounded by spontaneous changes in signs or symptoms that are unrelated to these therapies, between month 1 and 3 (therapy *A*) and between month 2 and 3 (therapy *B*). If a spontaneous change occurred in the same direction for both therapies (a continuous increase or decrease respectively), this change may be larger for therapy *A* with a post-treatment interval of 2 months than for therapy *B* with an interval of 1 month. If chance fluctuations were involved, the incidence of these may depend on the duration of the post-treatment interval and thus differ between therapies *A* and *B*. One might argue that chance fluctuations will be diminished by averaging across a patient sample. However, this diminution will be imperfect because patient samples have a limited size. Hence, spontaneous changes may cause artifactual differences between therapies in success rate or effectiveness when unequal post-treatment intervals are involved.

Thus allowing a therapy-and-patient-specific variation of treatment duration in RCTs will comply with clinical care, and enables an unbiased comparison of the therapeutic potential of different therapies. Such a type of RCT can be applied straightforwardly to non-life threatening disorders like TMD. In contrast to an RCT with variable treatment duration, a traditional RCT does further not provide information on the therapy duration and number of visits needed in individual patients. This information is of interest for a complete costs-effectiveness-analysis, and for attaining an optimal therapy strategy for myogenous TMD as outlined below.

The first aim of the present study was to evaluate treatment outcome of physiotherapy in comparison to that of splint therapy of myogenous TMD, using the index ‘Treatment Duration Control’ (TDC) for enabling an RCT with a therapy-and-patient-dependent number of visits and treatment duration [19]. All three features of treatment outcome have been addressed, *i.e.* (1) the time and number of visits needed to decide whether a patient’s treatment is either successful or unsuccessful, (2) success rate, and (3) therapy effectiveness (*cf.* Methods).

Whereas a multi-therapy modality is most appropriate for patients having TMD as well as major psychological problems, a single form of treatment each time is indicated for most TMD categories [20]. A single treatment modality could be applied, *i.e.* either physiotherapy or splint therapy, as patients with mainly basic signs and symptoms of myogenous TMD were selected (*cf.* Methods). However, like in common clinical care, an initial treatment became unsuccessful in a substantial fraction of the patients (*cf.* Results). Because myogenous TMD is a non-life threatening disorder, a trajectory of stepped-care is possible to increase the overall success rate. A trajectory starts with a first type of therapy which, if not successful, is followed by a second type. Patients whose physiotherapy was unsuccessful in the present study continued with splint therapy and reversely. The overall success rate of a trajectory may depend on (i) the therapies’ success rate when applied separately, (ii) their sequence of successive application, and (iii) a possible effect on the success rate of a subsequent therapy by the preceding one. The influence of these factors has been examined by using a theoretical stepped-care model.

The second aim of the study was to examine whether physiotherapy or splint therapy may be preferred as an initial treatment in stepped-care. To that end, all features of therapy outcome were considered as well as outcomes of the stepped-care model. A preliminary report has been published previously, in which patient samples and the validation of TDC were not completed yet [21].

## Methods

### Patients

The study was carried out in compliance with the Helsinki Declaration, and approved by the University Ethics Committee and the Board of Developmental Medicine in the Netherlands (reference: OG/93/002). The myogenous TMD patients were either referred to the Department in Utrecht (85%) or recruited directly from general dental and medical practitioners (15%). Recruitment, interventions and follow-ups occurred between April 1993 and March 2000.

**Fig. 1:**
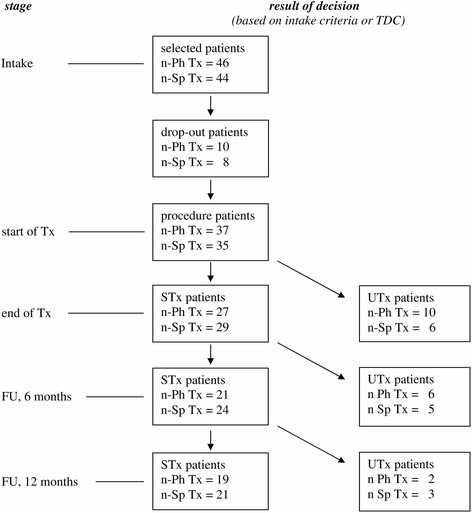
Patient flow. Tx, treatment. FU, follow-up evaluation, 6 and 12 months after end of Tx. Dropout patients, patients who did not complete the entire treatment procedure at various stages, for various reasons (see text); STx and UTx patients, patients whose Tx is successful or unsuccessful respectively, according to the TDC procedure, at a particular stage; n-Ph Tx, number of patients assigned to physiotherapy; n-Sp Tx, number of patients assigned to splint therapy

Out of 2078 patients, 187 patients (9%) were selected who had solely myogenous TMD, using stringent criteria. From these eligible patients, 37 patients (20%; nine males and 28 females) declined participation, mainly because of time and distance reasons. The remaining 150 patients gave informed consent of which 60 patients who had pronounced occlusal interferences participated in an RCT with other interventions (see below), and 90 patients without pronounced occlusal interferences enrolled in the present study. The current procedures were completed by 72 out of 90 patients, 37 patients for physiotherapy and 35 ones for splint therapy. Hence, 18 out of 90 patients (20%) became dropouts (ten patients for physiotherapy and eight patients for splint therapy, Fig. 1) at various stages of the procedure, for the following reasons: (1) the threshold of signs and symptoms was not met at the start of treatment (one patient assigned to physiotherapy), (2) problems with complying to appointments (work situation, distance), and (3) not able to complete treatment according to the protocol (medical co-intervention, co-morbidity, acute dramatic life events).

Assuming a mean baseline level of pain intensity of 30.6 mm on a 100 mm VAS for TMD [22], an SD of 19.0, and a reduction of the mean level of 35%, yielded an estimated sample size of 50 patients in each group to achieve a power of 0.80 at a level of type I errors of 5%. Considering the number of complying patients, the power has been calculated post-hoc for two types of outcome variables (*cf.* Results and Additional file 1, ‘Post-hoc power analysis on measures of effectiveness’).

As outlined in our previous study on pain patterns from the same patients [23], RDC/TMD [2] was followed to select group Ia and Ib patients (myofascial pain) while excluding group II (disk displacement of the Temporomandibular joint) and group III (arthralgia, arthritis, arthrosis). Patients with mainly basic signs and symptoms of myogenous TMD were selected, *i.e.* patients without possibly confounding influences from the temporomandibular joint, dental anomalies, major psycho-social factors, or factors affecting general health [23]. A specific exclusion criterion was previous treatment with an occlusal splint, occlusal adjustment, or physiotherapy of the masticatory system. Patients who had other treatments for pain (also orofacial pain) more recent than a year, were also excluded. Regarding major psycho-social factors, patients with a recent dramatic life event (details, see ref [23]) and/or psychotherapy and/or use of psycho-medication, were excluded to avoid a possible influence of extremely large levels and fluctuations of stress and/or an influence of types of medication, which might overshadow the treatment effect of physiotherapy or splint therapy. Hence, while stress which is related to common daily activities was still possible, the effect of physiotherapy or splint therapy on myogenous TMD may then be more related to myogenous and psychobiological factors. Table 1 shows pre-treatment values of demographic and clinical variables. The pre-treatment values of psychosocial variables, including those related to anxiety, depression, health locus of control and coping style have been reported previously [23].

**Table 1:** Demographic and clinical variables before the start of treatment

	Compliers		Dropouts
	Physiotherapy	Splint therapy	
Demography:			
Number of patients [n]	37	35	18
Age [mean, yrs (SD)]	31.4 (9.6)	29.0 (9.6)	28.7 (9.0)
Sex [female %]	95%	91%	94%
With partner [patient %]	56% *n = 36	40%	50%
Housekeeping [main responsibility, patient %]	58% *n = 36	58% *n = 31	53% *n = 15
Outdoors activity [work/study; patient %]	83% *n = 36	82% *n = 33	69% *n = 16
Both outdoors and housekeeping [patient %]	47% *n = 36	57% *n = 30	36% *n = 14
Clinical data:			
PM-patients [patient %]	69% *n = 36	82% *n = 33	80% *n = 15
Duration of PreTx pain [mean, months (SD)] *n = 17	25.6 (29.9)	18.2 (19.9)	27.3 (24.6) *n = 17
Duration of PreTx pain [median, months]	14	10	18 *n = 17
No spread of pain; only facial areas [patient %]	27%	34%	17%
Limited spread of pain; facial and neck areas [patient %]	5%	14%	11%
More extended spread of pain; facial, neck and shoulder areas [patient %]	68%	52%	72%
Predominant pain intensity, at the initial visit [mean, mm (SD)]	60.4 (22.4)	53.6 (13.1)	59.6 (18.8)
Predominant pain intensity, at start of Tx [mean, mm (SD)]	41.0 (23.4)	39.1 (22.5)	43.6 (20.2)
HR-QoL, EQ-5D [mean, utility value (SD)]	0.707 (0.202)	0.773 (0.176) *n = 32	*n = 0
Use of over-the-counter (OTC) medication:			
Patient %	54%	39% *n = 33	67% *n = 15
Percentage of possible times of scoring [%-value, mean (SD)]	7.1 (9.9)^§^	3.0 (5.2)^§^ *n = 33	16.5 (19.7)^†^ *n = 15

dropouts: *n* = 10 for physiotherapy and *n* = 8 for splint therapy. PM, Post Meridian patients with a maximal VAS-score of pain intensity at dinner or bed time, from a pain diary [23]. Spread of pain, data from the Pain Location Questionnaire [23]. HR-Qol, general Health-related Quality of Life using Euroqol-5D (EQ-5D). Use of over-the-counter medication, data from a pain diary [23]. *cases of missing values with indication of the actual number of patients (n). Differences between groups were only significant for the use of OTC medication, percentage of possible times of scoring. (^§^between complier groups, *p* < 0.05; ^†^dropouts vs. compliers, *p* < 0.05). All variables were obtained at the initial visit, except for ‘PM-patients’ and ‘use of OTC medication’ which were obtained during 2 weeks before the start of treatment, from a pain diary, and for ‘Spread of pain’ and ‘HR-QoL’ which were obtained just before the start of treatment

The aetiologic significance of occlusal interferences in the dentition has been questioned because of weak associations between such interferences and TMD in general [24]. However, apart from critical considerations on the dental literature about the relationship of occlusion and myogenous orofacial pain, some well controlled clinical and experimental studies are in favour of a possible aetiologic role of occlusal interferences in myogenous TMD [25]. Patients with pronounced occlusal interferences were therefore excluded in the present study for diminishing the influence of a possibly confounding factor, using as exclusion criteria: (1) a forward sliding of at least 2 mm and/or lateral sliding of at least 1 mm with respect to centric occlusion; and (2) an interference on the non-active side that is not accompanied with contact on the active side.

### Procedures between intake and start of treatment

At the initial visit, eligible patients were informed about a study on treatment effect. The project funded travel costs, costs of treatment if needed, and costs of, for example, baby-sitting for follow-up visits. All participants were informed in a standardized way about TMD as being a non-life threatening disorder with a lack of an unambiguous cause of the pain and about possible contributing factors. The patients received counselling on avoiding possibly stress-induced habits of grinding, clenching, nail biting or biting on objects like pencils, excessive gum chewing, biting and/or sucking on the lip or cheek, and pressing and/or sucking on the tongue. The patients were further informed that depending on the outcome of the final diagnosis, treatment would start at the second visit and would be based on one of four possibilities: (i) occlusal appliance, (ii) slight occlusal adjustment, (iii) a combination of occlusal appliance and slight occlusal adjustment, or (iv) physiotherapy of the masticatory system. The possibilities of ‘slight occlusal adjustment’ or its combination with ‘occlusal appliance’ were applied in another study (paper in preparation) in which myogenous TMD patients with pronounced occlusal interferences participated. In order to blind the patients and clinicians at the initial visit about the treatment allocation following randomization, dental impressions necessary to prepare dental casts for treatment options (i)-(iii) but not (iv) (physiotherapy), were obtained from all patients.

The patients of the present study were, using computer-generated random data, randomized by an independent researcher, across two therapies: (1) occlusal splint therapy, and (2) physiotherapy of the masticatory system (Fig. 1). Physiotherapy was the active treatment which was compared with a control treatment, occlusal splint therapy. Block randomization was used with an intended block size of 100 patients (90 were realized; Fig. 1) and an allocation ratio of 1:1:1:1 for 4 subgroups, *i.e.* (1.1) splint therapy for ‘younger’ patients (age ≤ median age of 32 years; expected median from ref [26]), (1.2) splint therapy for ‘older’ patients (age > 32 years), (2.1) physiotherapy for ‘younger’ patients, and (2.2) physiotherapy for ‘older’ patients. By considering these two age groups, a stratification occurred across the therapy groups for age and possibly related factors which might influence treatment success, such as duration of pre-treatment pain and, although not recent (>1 year), a previous treatment for pain. This stratification ensured a similar distribution of pre-treatment values from age-related factors in both patient samples which had a limited size (*n* = 35–37compliers; Fig. 1).

Evaluation of a patient’s status was carried out not only by the person who carried out treatment (the ‘clinician’, a dentist for dental therapies and a physiotherapist for physiotherapy), but also by an assessor (another dentist) who was blinded to the type of treatment and the patient’s medical history. Using data from the assessor, a third dentist, the investigator (author RG), determined the outcome values of TDC, to keep the assessor blinded. When a physiotherapist carried out treatment, a dentist who was responsible for the patient, carried out a final evaluation as ‘clinician’. Several clinicians and assessors were available, i.e. ten dentists and five physiotherapists as clinician and four dentists as assessor. All abovementioned persons were specialists in treatment of orofacial pain and TMD, and were calibrated using a pilot group of 20 patients, which was also used for tuning the cut-off value of TDC (−0.379, see below) for distinguishing between a successful treatment and an unsuccessful one [19]. Following randomization for therapy, each patient was independently assigned to a particular clinician for treatment and a blinded assessor for the entire procedure. The patients were approximately stratified across the participating clinicians and assessors.

A similar waiting time between the initial visit and start of treatment occurred for both types of therapy, *i.e.* on average 4.4 weeks (SD 2.6) for splint therapy and 4.4 weeks (SD 2.4) for physiotherapy. The waiting time was at least 2 weeks to enable the scoring of a 2-week pain diary [23], and the preparation of a maxillary, flat-plane, hard acrylic occlusal appliance (Michigan type) [27], for those patients who were assigned to splint therapy.

### General procedure of treatment and outcome

The score profile of the patients was determined using data from an anamnestic and clinical examination. The anamnestic questionnaire included scoring on adjectival 0–4 point scales of five items which were characteristic for mygenous TMD (for details, also for the clinical examination, see Appendix in ref [19]). The questionnaire also included scoring of the intensity of the predominant pain from the masticatory system on a 100 mm Visual Analogue Scale (VAS; anchor points: ‘no pain’ and ‘the most intense pain one can imagine’). The clinical examination included scoring of pain intensity on an adjectival 0–4 point scale during jaw movements, palpation of jaw muscles, and during clenching. The total number of clinical items was 42. Regarding a threshold of signs and symptoms at the initial visit and the start of treatment, see Additional file 1, ‘Threshold of signs and symptoms’.

Each therapy had a specific program with a number of visits which could vary depending on the rate of a patient’s improvement (see below, section ‘Specific treatment procedures, general features’). Hence with an inter-visit interval which was therapy-specific, the duration of treatment could vary. The progress and ultimate effect of treatment were evaluated using the index ‘Treatment Duration Control’ (TDC). Details of the TDC procedure (including the use of added reference items), and on the validation of TDC can be found elsewhere [19]. Only some main features are presented below.

Baseline scores from anamnestic and clinical items were obtained by a blinded assessor, just before treatment and transferred by the investigator to keep the assessor blinded. This baseline assessment occurred on average 4.4 weeks (SD 2.5) following the initial visit. Using Smallest Detectable Difference (SDD) as a threshold, items with significantly large score values were selected as ‘reference items’ for monitoring relative change using the index TDC during treatment (by the clinician) and during follow-up (by the investigator, based on data from the blinded assessor).

The clinician carried out the anamnestic and clinical examination of the patient during the various treatment visits. The relative change in each reference item, between a later visit and the reference visit, was expressed as a contrast value, being the ratio between the difference and the sum of both score values of the reference item. The index TDC is the mean across all contrast values from the various reference items. TDC values vary within a range from +1.000 to −1.000, in which 0 represents no change, −1.000 represents complete recovery and a positive value represents worsening.

The clinician’s decision to continue or end treatment, thus controlling treatment duration, was based on two cut-off points of TDC. Each cut-off point corresponds to a global relative decrease of the scores of reference items. The first cut-off point was TDC = −0.212, which corresponds to a decrease of 35% in a single score of pain intensity at a 100 mm VAS. A less negative value than -0.212 means less change towards recovery. If, at a critical stage of treatment, a patient’s TDC was larger than −0.212 (less negative, more to zero), the patient was insufficiently responsive to treatment. The second cut-off point, TDC = −0.379, was related to attaining functional status for myogenous TMD (potentially ‘successful’ treatment with a residual level of signs and symptoms), and corresponds to 55% decrease of a single score of pain intensity. Depending on the TDC-outcome, the clinician continued or finished treatment within the limits of possible number of visits and their therapy-specific intervals. If TDC was > −0.212 after a therapy-specific minimum number of treatment visits, the treatment was ended because the patient was not sufficiently responsive. If −0.379 < TDC ≤ −0.212, a patient was sufficiently responsive but the treatment was continued as long as the maximal number of visits was not exceeded. If TDC was ≤ −0.379 at two successive visits, treatment was ended as being potentially successful.

Patients whose treatment was potentially successful or unsuccessful according to the findings of the clinician, were transferred to the assessor for blinded evaluation. The assessor carried out the anamnestic and clinical examination, on average 3.6 weeks (SD 6.0) after the end of treatment for all patients. The waiting time between the end of treatment and the first post-treatment visit for blinded evaluation was at least 2 weeks to enable the scoring of a 2-week pain diary [28]. For ethical reasons, patients with an unsuccessful treatment in the short-term, according to the data from the assessor, had no follow-up. Their initial unsuccessful treatment was immediately followed by another treatment (stepped-care). The other patients (successful treatment in the short-term) had a follow-up of 6 months and the follow-up was continued for another 6 months (thus 12 months follow-up in total) for those patients whose treatment was still successful after 6 months. The investigator determined post-treatment TDC-values for each patient using solely data from the blinded assessor.

Following the introduction of all score values in a custom-made spreadsheet (Microsoft Excel®; available on request) the reference items were automatically detected and all TDC-values were automatically determined for each patient.

### Specific treatment procedures, general features

For each type of treatment, a usual bandwidth of possible visits and their intervals was defined *a priori*. For splint therapy, the adaptive program (Additional file 1, ‘Rules for progressing and ending splint therapy’) could result in a number of visits and a treatment duration which varied within a range of 3–6 (visits) and 12–30 weeks (duration). For physiotherapy (Additional file 1, ‘Rules for progressing and ending physiotherapy’), the possible number of visits varied within a range of 10–16 and the treatment duration within a range of 10–21 weeks. Although the visit program was respected as much as possible, like in usual clinical care, this program could somewhat be adapted in view of holidays, illness or limitations of appointment opportunities. The rules from the Additional file 1 for progressing and ending therapy were transformed to decision trees to provide an overview of a patient’s treatment to the clinician.

### Specific treatment procedures, splint therapy

For patients who were assigned to splint therapy, the occlusal appliance (Michigan type) was applied in the upper jaw, and the patient was instructed to wear the splint as much as possible, at least in the evening and overnight for a minimum of 10 to 12 h. During treatment, the clinician determined the patient’s status every 6 weeks by anamnestic and clinical examination and by determining TDC. Patients could, if needed, have a short interim visit for minor adjustment of the splint.

As soon as the patient’s signs and symptoms decreased sufficiently, as indicated by TDC ≤ −0.379, the splint was gradually withdrawn during the forthcoming 6 weeks, *i.e.* by wearing the splint for 6 nights during the first week of withdrawal, five nights during the second week etc. If the patient’s signs and symptoms remained sufficiently low, thus if TDC ≤ −0.379 occurred at two successive visits corresponding to an entire period of 12 weeks, the clinician considered the splint therapy as being potentially successful and the patient was referred to the blinded assessor who applied the abovementioned outcome procedure. Otherwise, the clinician ended the splint therapy as unsuccessful and such a patient was also referred to the assessor. Patients whose splint therapy was ended successfully were allowed to apply splint wearing again if they felt a need.

### Specific treatment procedures, physiotherapy

The aim of physiotherapy was learning techniques to (1) avoid stress related pain from the masticatory system and (2) relieve this pain by means of self-massage and relaxation. During the first 3 weeks, all patients participated 2–3 times a week in an intensive program with instructions and exercise regarding (on indication): (i) posture of head, neck, shoulders, jaw and tongue, and (ii) opening movement of the jaw, with control of rotation and translation, (iii) progressive relaxation of jaw muscles using the method of Jacobson,(iv) counselling on avoiding excessive jaw opening, and habits like biting on objects and unilateral chewing, (v) pain relieve by means of self-massage of sore or painful facial and/or jaw muscles, (vi) stretching of jaw-closing muscles intra-orally by using the thumb, and resisting jaw-opening by placing the hand under the chin, by which jaw-closing muscles are relaxed while jaw-opening muscles are activated, (vii) habit-reversal techniques for avoiding habits like nail-biting, lip biting, biting on, for example, pencils, clenching or grinding, or sucking on the tongue and (viii) mostly at the end of the program, enhancement of the capacity of loading the muscles by chewing different types of foods and chewing gum.

Following the basic program of 3 weeks, patient-specific exercises were continued at home for 6 weeks, for those patients whose signs and symptoms has decreased sufficiently, as indicated by TDC ≤ −0.379. Otherwise, a patient-specific program was continued with weekly controls with a maximum of 6 weeks (for details, see Additional file 1, ‘Rules for progressing and ending physiotherapy’). As soon as TDC ≤ −0.379 was attained at such a control, home exercise was carried out for 6 weeks. Following 6 weeks of home exercise the patient was clinically examined by the responsible dentist (who acted then as ‘clinician’). If TDC ≤ −0.379, the physiotherapy was considered as being potentially successful and the patient was referred to the blinded assessor who applied the outcome procedure. Otherwise, the patient returned to the physiotherapist for one additional period of home exercise during 6 weeks after which final clinical evaluation was carried out. Patients whose physiotherapy was ended successfully were allowed to apply home exercise again if they felt a need.

### A stepped-care model

Using stepped-care, patients whose splint therapy was unsuccessful continued with physiotherapy and reversely. In order to limit the project duration, the subsequent treatment was part of common care and thus not controlled by TDC. However, a theoretical stepped-care model has been developed to assess the overall success rate of a trajectory of one therapy or two successive therapies if stepped-care is necessary (for details, see Additional file 1, ‘A stepped-care model including two possible therapies’) [29].

The overall percentage success rate of a trajectory (*SR*_*tr*_) of a therapy *A* which is possibly followed by a second therapy *B*, is given by:

*SR*_*tr*_ = (*f*_*A*,*S*_ + *m. f*_*B*,*S*_ – *m. f*_*A*,*S*_. *f*_*B*,*S*_)*.100* % (equation (6) in Additional file 1), in which *f*_*A,S*_ and *f*_*B,S*_ are fractions of patients for which therapy *A* and *B* respectively are successful when applied separately (thus without a preceding therapy *A* when therapy *B* is applied). Each of these fractions equals the basic percentage success rate (*SR*_*A*_ or *SR*_*B*_) divided by 100. *m* is a modulation factor which describes the possible influence of a preceding therapy *A* on the basic success rate of therapy *B* (*m ≥ 0*).

### Outcome variables of the RCT

The primary outcome variables were: (i) number of visits/duration of treatment used which is based on TDC during treatment, (ii) success rate based on post-treatment TDC, and (iii) effectiveness based on post-treatment TDC. While number of visits/duration of treatment were known at the end of treatment, success rate and effectiveness were determined following treatment, *i.e.* after on average 3.6 weeks, and for patients whose treatment was successful in the short-term, also after 6 and 12 months. Weighing of these primary outcomes enabled a decision on whether physiotherapy or splint therapy may be recommended as an initial treatment of myogenous TMD in stepped-care.

Success rate was determined for each therapy group using the criterion TDC ≤ −0.379. Furthermore, the mean and SD of the post-treatment TDC values were determined for each therapy group for comparing therapy effectiveness in terms of relative decrease in scores from significantly pronounced signs and symptoms which are patient-specific [19]. Mean TDC-values from the last post-treatment visit were used as a measure of effectiveness. Four out of 72 patients whose treatment was successful in the short-term (three patients for splint therapy and one patient for physiotherapy) could not be reached for the 1-year follow-up, although their treatment was successful at an earlier post-treatment stage, *i.e.* following treatment (one patient) or after a follow-up of half a year (three patients). Using an intent-to-treat analysis, the last post-treatment observation was carried forward thus missing values during the follow-up were replaced with the last previous non-missing value.

Because of a lack of comparable outcome TDC-values to date, the intensity of predominant pain of the masticatory system has been used as a key parameter to compare therapy effectiveness of the present study with that from other studies (*cf.* Discussion). This secondary outcome variable was determined at the initial visit (on average 4.4 weeks before the start of treatment), at the start of treatment, and following treatment, *i.e.* on average after 3.6 weeks, and for patients whose treatment was successful in the short-term, also after 6 and 12 months. Pain intensity has been analysed in a traditional manner, *i.e.* by comparing its pre- and post-treatment values and by determining effect size (Cohen’s *d*) for each therapy using an online effect size calculator [30]. The change in the mean of raw scores of an outcome variable observed after an intervention of known effectiveness is an estimate of Clinically Important Difference (CID) [31]. In order to characterize the effect of interventions in general, this change, normalized as a percentage of the scale range (scale-% units), was denoted as the Clinical Difference (CD). Cohen’s *d* is the ratio between the non-normalized CD and the pooled SD of the scores from two times of measurement. Cohen’s *d* was bias corrected [32]. Values of *d* between 0.20 and 0.49 represents a small effect of treatment, those between 0.50 and 0.79 a medium effect, and those equal to or larger than 0.80 correspond to a large effect.

As the initial treatment of a substantial fraction of the patients became unsuccessful (*cf.* Results), it was explored whether some subgroups of myogenous TMD patients may respond better to physiotherapy or splint therapy than other ones. Possible differences in pre-treatment values of variables were therefore examined between groups for which treatment became successful or unsuccessful respectively. To that end, variables were used with gradual levels for which the number of patients in the sub-samples was still fairly large (*n* = 14–21). Apart from the demographic variable ‘age’, these differences were examined for the clinical variables, ‘duration of pre-treatment pain’, ‘pain intensity’, ‘Health-related Quality of Life’ and ‘number of times of use of OTC-medication’ (Table 1). Furthermore, such possible differences were examined for 20 psychosocial variables, including those related to anxiety, depression, health locus of control and coping style (*cf.* Table 4 in ref [23]).

### Statistical analysis

Statistical analyses were performed using Graphpad software (Graphpad Prism 6.04; Graphpad Software Inc., San Diego, CA). Differences in frequency between two patient groups were examined using a squared-Chi test or Fisher’s exact test. A Student’s t-test for paired observations was used for examining intra-subject differences in values of continuous variables from two times of measurement, and a Student’s t-test for unpaired observations for inter-subject differences in values obtained at one occasion. The values of ‘duration of pre-treatment pain’ which were not normally distributed and positively skewed, were first log transformed. One-way ANOVAs were used when four patient subgroups were involved in one factor, for example, post-treatment TDC-value. Because the variance of individual TDC values depends on the number of contributing items [19], it was examined whether the distribution of contributing items was similar between different patient groups for enabling an unbiased statistical comparison between group means of TDC. Two-ways ANOVAs were used when two factors with 2–4 levels were involved, i.e. stage of treatment (four levels, paired observations) and either type of therapy or success outcome (two levels, unpaired observations). When a two-way ANOVA was significant at a level of 2.5% (Bonferroni correction of a 5% significance level for the two-fold use of data in these ANOVAs), Bonferroni’s multiple comparison tests were used to determine significance of differences between pairs of conditions. The level of significance was 5% otherwise. A u-test was used for examining whether mean post-treatment TDC-values differed from zero and were more negative, indicating an improvement of signs and symptoms at a group level.

## Results

Table 1 shows the pre-treatment values of demographic and clinical variables. Except for the small percentage of times of using OTC-medication (3.0–7.1%), significant differences of the other 16 variables did not occur between the two therapy groups of patients who completed the entire procedure, and between the compliers and the dropouts. When patients who did not use OTC-medication were excluded in the analysis, the percentage of times of using this medication only tended (*p* = 0.059) to be larger for patients who completed the procedures for physiotherapy rather than for splint therapy (physiotherapy: 13.1% of the possible times, SD 10.1, *n* = 20; splint therapy: 7.7%, SD 5.7, *n* = 13).

Table 2 shows that the success rate (SR) of physiotherapy was similar to that of splint therapy, in the short-term (at EM: 73–83% of the patients) as well as in the long-term (LM: 51–60%). The Relative Risk (RR: a probability of which its value equals the ratio between SRs from physiotherapy and splint therapy) was 0.88 at EM (95% confidence interval: 0.69–1.13) and 0.86 at LM (95% confidence interval: 0.57–1.30). Hence, patients treated with physiotherapy were 12% (EM) and 14% (LM) respectively less likely to have a successful treatment rate than with splint therapy, but this is not statistically significant. The SR outcomes were attained with a significantly (*p* < 0.001) shorter treatment duration for physiotherapy (on average 10.4 weeks less) and, on the other hand, a significantly (*p* < 0.001) smaller number of treatment visits for splint therapy (on average 7.1 visits less).

**Table 2:** Success rate, treatment duration and number of visits

	Phyiotherapy	Splint therapy	*P*-value and significance of difference
Number of patients	37	35	
SR (% patients) at EM	73%	83%	0.339 NS^†^
SR (% patients) at LM	51%	60%	0.487 NS^†^
Duration of treatment [mean, weeks (SD)]	13.8 (6.5)	24.2 (9.2)	*p* < 0.0001^‡^
Number of visits [mean, (SD)]	11.5 (2.0)	4.4 (1.1)	*p* < 0.0001^‡^

SR, success rate. EM, end-measurement of treatment outcome in the short-term, at the first post-treatment visit. LM, last measurement of treatment outcome in the long-term, at the last post-treatment visit. LM only includes an entire follow-up of 1 year for patients whose treatment continues to be successful from EM. number of visits: from the first visit of treatment (thus excluding the initial visit with intake) to the visit with the last control by the clinician included. ^†^ squared-Chi test. ^‡^ Student’s t-test for unpaired observations

Table 3 shows that the TDC-values in the long-term (therapy effectiveness) were similar for physiotherapy and splint therapy, regardless of the patient group (all patients, or patients whose treatment was successful or unsuccessful). The mean TDC-values were always significantly (*p* < 0.001–0.01) smaller (more negative) than zero, even for patients whose treatment was unsuccessful indicating improvement of signs and symptoms in these patients.

**Table 3:** Post-treatment TDC values in the long-term

	Physiotherapy	Splint therapy	*P*-value and significance of difference
All patients:			
TDC at LM [mean (SD), n]	−0.512 (0.339), 37	−0.575 (0.361), 35	0.446 NS^†^
Number of items contributing to TDC at LM [mean (SD), n]	14.2 (6.6), 37	15.7 (8.3), 35	0.407 NS^†^
Patients with STx:			
TDC at LM [mean (SD), n]	−0.807 (0.127), 19	−0.820 (0.161), 21	0.808 NS^‡^
Number of items contributing to TDC at LM [mean (SD), n]	12.2 (5.9), 19	15.6 (9.2), 21	0.152 NS^§^
Patients with UTx:			
TDC at LM [mean (SD), n]	−0.200 (0.161), 18	−0.208 (0.244), 14	0.906 NS^‡^
Number of items contributing to TDC at LM [mean (SD), n]	16.3 (6.8), 18	15.7 (7.0), 14	0.832 NS^§^

TDC at LM, last measurement of treatment outcome in the long-term. STx and UTx, successful and unsuccessful treatment respectively. ^†^Student’s t-test for unpaired observations. NS, non-significance. ^‡^one-way ANOVA for the factor TDC between the various patient groups with different therapies and treatment outcomes. The factor TDC was significant (*p* < 0.0001), indicating TDC-values which were smaller for patients with STx (more negative TDC-values indicating more improvement) than for patients with UTx. The Bonferroni’s multiple comparison tests were non-significant between both therapies, for STx and UTx respectively (*p*-values indicated). ^§^one-way ANOVA for the factor number of items contributing to TDC at LM which was non-significant (*p* = 0.328)

As a possibly unintended effect, new signs and symptoms of myogenous TMD could appear during a patient’s treatment, which did not occur at the start of treatment. If significant, the scores of such signs and symptoms were included in added reference items of the TDC-procedure [19]. The frequency of cases with added items during treatment was similar between physiotherapy (11 out of 37 patients; 30%) and splint therapy (11 out of 35 patients; 31%). For both types of therapy, added items occurred significantly (*p* < 0.05) less frequently in patients whose treatment became successful in the long-term than for patients with an unsuccessful treatment. While only 10% of the patients (two out of 19) whose physiotherapy was successful had added items, this frequency was 50% (nine out of 18) for the patients with unsuccessful physiotherapy. For splint therapy, these frequencies were 14% (three out of 21 patients) and 57% (eight out of 14 patients) respectively. However, the occurrence of added items never yielded cases of an interrupted treatment.

The post-hoc power of the present study to detect inter-therapy differences in mean TDC values is fairly large, *i.e.* 68% for detecting a TDC-difference of 35.0 and 80% for detecting a difference of 40.0%. These detectable differences in multidimensional TDC-values are equivalent to changes of 19.7–29.7% in the difference of a single variable, like ‘pain intensity’, before and after treatment (*cf.* Additional file 1, ‘Post-hoc power analysis on measures of effectiveness’).

Table 4 (top) shows that the intensity of the predominant pain from the masticatory system decreased significantly (*p* < 0.001; stage effect in a 2-way ANOVA) in the long-term. This decrease was similar for both therapies (*cf.* Additional file 1: Table S6, ‘Two-way ANOVA statistical analysis for pain intensity’). The decrease in the means of pain intensity between the start of treatment and the last post-treatment measurement, the Clinical Difference (CD), was 21.8 scale-% for physiotherapy and 27.6 scale-% for splint therapy. These CD-values corresponded to Cohen’s *d*-values of 0.86 and 1.39 respectively (Table 4, bottom).

**Table 4:** Predominant pain intensity from the masticatory system at three stages

Pain intensity per therapy group:					
Physiotherapy (*n*=37)			Splint therapy (*n*=35)		
Initial visit	Start-Tx	LM	Initial visit	Start-Tx	LM
[mean (SD)]			[mean (SD)]		
60.4 (22.4)	41.0 (23.4)	19.2 (26.4)	53.6 (13.1)	39.1 (22.5)	11.5 (16.2)
Cohen’s *d* between stages, per therapy group:					
		Physiotherapy		Splint therapy	
		*d*	Confidence interval (95%)	*d*	Confidence interval (95%)
Initial visit vs start-Tx		0.84	0.36–1.31	0.77	0.29–1.26
Start-Tx vs LM		0.86^a^	0.39–1.34	1.39^a^	0.87–1.92
Initial visit vs LM		1.66^b^	1.13–2.19	2.83^b^	2.17–3.49

Top: mean pain intensity in mm on a 100 mm VAS and SD (between brackets) for predominant pain at three stages: initial visit, start of treatment (Start-Tx) and at the last post-treatment visit, last measurement (LM). Both therapy groups have a similar significant stage effect (*p*<0.0001; 2-way ANOVA, *cf.* Additional file 1: Table S6). All inter-stage differences are significant (*p*<0.0001-0.01) in Bonferroni’s multiple comparison tests

Bottom: *d*, Cohen’s effect size based on pooled SD (*d*=(|mean_2_-mean_1_|)/SD_pooled_, in which ‘2’ refers to the later stage and ‘1’ to the earlier one), and bias corrected (Hedges). For the values of the means, see top. ^a,b^ significant differences of *d* between therapy groups as the means are mutually excluded from the confidence interval of the other therapy group

For a post-hoc power analysis regarding pain intensity, individual difference values were considered between the last post-treatment measurement and the start of treatment (*cf.* Additional file 1, ‘Post-hoc power analysis on measures of effectiveness’). The power is 39% for detecting an inter-therapy difference of 35.0% in the mean difference value of pain intensity, and 80% for detecting a difference of 58.4%.

Even before treatment, between the initial visit with counselling and the start of treatment, pain intensity decreased significantly (*p* < 0.0001–0.01) and similarly for both therapy groups (Table 4, top). CD was 19.4 and 14.1 scale-%, and Cohen’s *d* was 0.84 and 0.77 for patients assigned to physiotherapy and splint therapy respectively (Table 4, bottom).

Whereas Cohen’s *d* of decrease in pain intensity between the start of treatment and the last post-treatment measurement, was significantly smaller for all patients with physiotherapy than for those with splint therapy (0.86 vs. 1.39; Table 4 bottom), these *d*-values were similar (2.07 vs. 2.02) for patients whose treatment was successful. For patients whose treatment was unsuccessful, the *d*-values were similar as well, *i.e. d* was 0.39 for physiotherapy (95% confidence interval: −0.27–1.05) and *d* was 0.73 for splint therapy (95% confidence interval: −0.04–1.49).

**Fig. 2:**
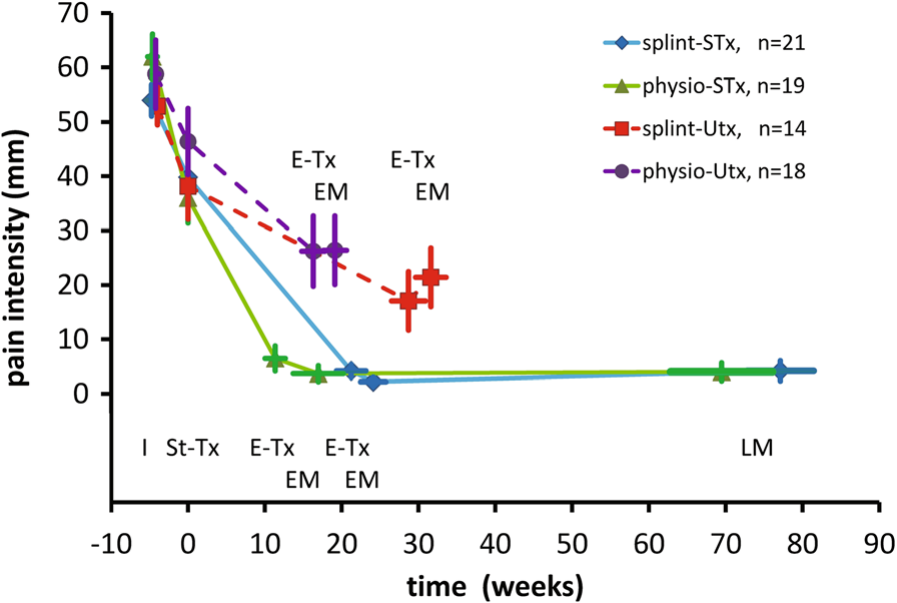
Intensity of predominant pain in the masticatory system. This intensity (mm on a 100 mm VAS) is depicted as a function of time for two types of therapy, two treatment outcomes according to TDC (successful treatment, STx; unsuccessful treatment, UTx), and the various pre-treatment and treatment stages. Mean and SEM are depicted for pain intensity as well as the timing of the stages. Stages: I, initial visit; St-Tx, start of treatment (corresponding with the zero point of time); E-Tx, end of treatment; EM, end measurement of treatment at the first post-treatment visit; LM, last measurement from patients with a successful treatment, following a 1-year-follow-up. For statistical testing of the various levels of pain intensity, see Additional file 1: Table S7

For both therapy groups, Fig. 2 shows the pain intensity as a function of time for the two subgroups of patients, *i.e.* patients whose treatment was successful (STx) or unsuccessful (UTx) in the long-term. Figure 2 depicts a decrease in pain intensity between initial visit and start of treatment, for the four subgroups of patients. Physiotherapy and splint therapy attained similar end levels of pain intensity which were significantly higher in patients with UTx than in patients with STx (see Additional file 1: Table S7, ‘Two-way ANOVA statistical analysis for pain intensity’). The time needed to attain these similar end levels was shorter for physiotherapy than for splint therapy. Regardless of the type of therapy, pain intensity not only decreased significantly between start and end of treatment in patients with STx but also in patients with UTx. In patients with UTx, this decrease halted at a higher end level than that of patients with STx. For patients with UTx, the higher level at the end of treatment, stage E-Tx, was similar to the post-treatment level at EM (Fig. 2), on average 3.6 weeks later. For both types of therapy, pain intensity of patients with STx was similarly small at the post-treatment stages EM and LM (Fig. 2; no significance in a Student’s t-test for paired observations).

In order to explore which factors might decrease a patient’s responsiveness to a therapy, the baseline levels of several factors have been compared between patients whose treatment became successful and unsuccessful respectively. Regardless of splint therapy or physiotherapy, significant differences between both patient groups did not occur for the various clinical variables examined, including ‘number of times of use of OTC-medication’, which differed between the entire therapy groups at baseline (Table 1). It is therefore notable that the fraction of patients who used this medication was not significantly related to treatment outcome (physiotherapy: eight out of 19 patients with a successful treatment (42%), used OTC-medication, and 12 out of 18 patients with an unsuccessful treatment (67%); splint therapy: ten out of 21 patients with a successful treatment (47%), and three out of 14 patients with an unsuccessful treatment (21%)).

Significant differences were also, in general, absent for the various psycho-social variables examined. Only one out of 20 psycho-social variables differed significantly (*p* < 0.05), *i.e.* internal locus of control for physiotherapy which was 4.74 (SD 0.81, *n* = 19; questionnaire ‘Multidimensional Health Locus of Control’, MHLC [23]) for patients whose treatment was successful and 3.94 (SD 1.20, *n* = 17, 1 missing value) for patients with an unsuccessful treatment.

Physiotherapy and splint therapy were considered in the stepped-care model. Table 5 shows the success rate of each of the two possible trajectories, *SR*_*tr*_, for three values of the factor *m*, which modulates the basic success rate of the second possible therapy in a trajectory, thus describing the influence from the preceding therapy. For *m = 1* (no change in success rate of the subsequent therapy), *SR*_*tr*_ is 80.5% of the patients, regardless of the sequence in which the two therapies are applied. For *m = 0.5* (halving the basic success rate of the subsequent therapy), *SR*_*tr*_ is 10.2–14.7 patient-% units smaller than *SR*_*tr*_ for *m = 1*. The largest value of *SR*_*tr*_ occurs with *m = 0.5* when the trajectory is started with splint therapy for which, although not significant, the observed value of the basic success rate is larger than that for physiotherapy, *i.e.* 60% vs 51% (Table 2). However, the difference in success rate between the two possible trajectories with reversed therapy sequences, 4.5%, is smaller (half in this example) than the difference, 9%, in basic inter-therapy success rate (*cf.* equation (11) in Additional file 1, ‘A stepped-care model including two possible therapies’). For *m = 1.5* (enhancing the basic success rate of the subsequent therapy by this factor), *SR*_*tr*_ is 10.2–14.7 patient-% units larger than *SR*_*tr*_ for *m = 1*, and, in contrast with *m = 0.5*, the largest value of *SR*_*tr*_ occurs when the trajectory is started with the therapy with the smaller value of success rate, hence physiotherapy. The difference in success rate between the two possible trajectories, 4.5%, is, like for *m = 0.5*, also smaller than the difference in basic inter-therapy success rate.

**Table 5:** Success rate in stepped-care of trajectories consisting of one or two therapies

	Trajectory		
	Physiotherapy possibly followed by splint therapy	splint therapy possibly followed by physiotherapy	difference between trajectories
*SR*_*tr*_ for *m = 1* (patient %)	80.4%	80.4%	0.0%
*SR*_*tr*_ for *m = 0.5* (patient %)	65.7%	70.2%	4.5%
*SR*_*tr*_ for *m = 1.5* (patient %)	95.1%	90.6%	4.5%

*SR*_*tr*_, success rate of a trajectory. A trajectory consists of a first therapy which is possibly followed by a second therapy if the first one is unsuccessful. *SR*_*tr*_ has been calculated for the two possible sequences of physiotherapy and splint therapy, according to the stepped-care model (equation (6) in Additional file 1, ‘A stepped-care model including two possible therapies’), using the basic success rates of these therapies (*cf.* Table 2, at stage LM) and three values for the modulation factor *m*, which reflects the degree by which the success rate of the second therapy is diminished (m < 1) or enhanced (m > 1)

## Discussion

### Effect of Counselling on Pain Intensity

Like in a traditional RCT, stringent inclusion and exclusion criteria were applied to select myogenous TMD patients. In contrast to previous RCT studies on myogenous TMD patients [14, 15, 18, 33, 34], the entire treatment procedure using TDC was similar to one in general clinical care. This procedure included counselling, a therapy-and-patient-specific duration of treatment, and possibly intermediate adjustment of an occlusal splint. Furthermore, patients whose therapy was successful were allowed to apply splint wearing or physiotherapy (home exercise) respectively when they would feel a need. The influence on pain intensity of the standardized reassurance and counselling which preceded both types of therapy, will first be discussed, followed by the influence of treatment on pain intensity. The post-treatment TDC-values will be discussed thereafter.

The intensity of the predominant pain from the masticatory system decreased on average 32% between the initial visit (100%) and the start of treatment with a CD of 16.6 scale-%. The value of Cohen’s *d*, on average 0.80, reflects a moderate/large effect of counselling on pain intensity. Pain intensity was constant in a pain diary, during 2 weeks before the start of treatment [23]. Hence, the decrease of pain intensity occurred shortly after counselling rather than late in the waiting period of 4.4 weeks.

Regarding other studies, in a heterogeneous sample of TMD patients [35], pain intensity decreased similarly by 34% (CD: 22.8 scale-%), with an effect size *d* of 1.13 (mean and SD values of *d* derived from a Figure), following counselling and a waiting period of 2 weeks. A similar decrease of 15.4% (CD 10.1 scale %, *d*: 0.73) of pain intensity was observed 1 month following initial visit and solely counselling, during maximum unassisted jaw opening in myogenous TMD patients whose jaw opening was limited (RDC/TMD: class 1b) [36].

Because of a general association with pain and the decrease of pain intensity following counselling, the signs and symptoms of one patient assigned to physiotherapy decreased below the threshold following the initial visit, so that this patient became a dropout. Hence, for patients with a low level of signs and symptoms at the initial visit, treatment may be restricted to counselling and instructing simple stretch and auto-massage techniques. A future application of the TDC-procedure from the initial visit will be of interest to determine objectively for which patients counselling and education will be sufficient to attain functional status.

### Effect of Treatment on Pain Intensity

Because counselling at the initial visit causes a fast decrease in pain intensity which stabilizes, a further decrease must be due to the applied therapy, rather than to a natural course of the disorder. Cohen’s *d* was 0.86 and 1.39 for physiotherapy and splint therapy respectively. Thus the effect on pain intensity of these therapies which were controlled by the TDC-procedure is large (*d* > 0.80). These *d*-values and the CD-values (21.8–27.6 scale-%) are similar to those from previous studies on mainly myogenous TMD patients without overt psychosocial factors, regardless of the type of pain of which the intensity was considered. In these previous studies [14, 15, 33, 34, 37], the effectiveness of either physiotherapy or splint therapy has been examined using a constant treatment duration between 5 and 10 weeks, and treatment was preceded or combined with counselling including reassurance of the patients. Following splint therapy, CD of maximal pain was 23.2 scale-% (final evaluation after 5 weeks, active splint group) [33] and 28.8 scale-% (12 months) [14, 15] and *d* was 0.99 and 1.48 respectively. Following physiotherapy, CD of present pain was 22.5 scale-% for intra-oral physiotherapy alone and 34.9 scale-% for a combination of such physiotherapy and education (12 months) [34]. In another study on physiotherapy (6 weeks, combined groups) [37], CD was 25.4 scale-% for maximal pain and *d* was 1.35. The number of treatment visits (10–18) in the previous studies on physiotherapy was similar to the ones in the present study (mean 11.5 visits, SD 2.0). The number of treatment visits for splint therapy was five in the study of Raphael and Marbach [33] which is similar to the mean one in the present study (4.4 visits), and at least two visits in the study of Ekberg et al. [15]. The number of visits from the previous studies on splint therapy does not include additional visits for a withdrawal of wearing the splint when splint therapy became successful. Treatment durations of 5–10 weeks in these studies are therefore a lower limit of a realistic duration.

The abovementioned findings show that when splint therapy is preceded or combined with counselling including reassuring the patients, this therapy has a large effectiveness which is similar to that of physiotherapy from separate studies. However, without such counselling, there was no effect of splint therapy on pain intensity [18]. Following 3 months of splint therapy (6 visits with only splint control), CD for intensity of spontaneous muscle pain was −2.8 scale-% and Cohen’s effect size (ES: ratio between difference in means and baseline SD) was merely −0.14 (negative sign: slight worsening). An effect of solely splint therapy was also lacking for pain intensity during gum chewing [18], *i.e.* CD was 3.4 scale-% and ES was 0.11. The effect of solely counselling and education which was repeated 6 times during 3 months was moderate for intensity of spontaneous muscle pain [18], *i.e.* CD was 11.2 scale-% and ES was 0.58 and small for pain intensity during gum chewing (CD: 7.0 scale-%, ES: 0.30). Thus all findings suggest that a synergy occurs between counselling and splint therapy, *i.e.* absence of counselling blocks the potential effect of splint therapy and/or splint therapy enhances the effect of counselling. Because the effect of a one-time counselling at the initial visit was stabilized in the present study before splint therapy was started, blocking of the effect of splint therapy is probably involved anyhow.

The small/moderate effect of solely counselling during 3 months was reflected in a relatively large VAS-score of intensity of spontaneous muscle pain (30 mm on a 100 mm VAS) or pain following chewing (38 mm) at the end of the program [18]. Such end levels of pain intensity, which are even higher than the mean ones for patients whose treatment was unsuccessful in the present study (Fig. 2), are much higher than that of patients with a successful treatment, who have attained functional status (mean pain score < 7 mm; Fig. 2). Hence, solely counselling and education will, in general, not be sufficiently effective to attain functional status within a reasonable time. This conclusion is reinforced by findings from another study on myogenous TMD patients (RDC/TMD: class 1b) [34]. Although monthly counselling continuously decreased the intensity of pain at maximum unassisted mouth opening, this intensity was still large following 3 months (43 mm) and 12 months (27 mm). The large effectiveness which was observed for physiotherapy in the present study might in part be due to a repeated education procedure which is inherently part of this type of physiotherapy.

In the present study, Cohen’s *d* of pain intensity was significantly larger for splint therapy (*d* = 1.39) than for physiotherapy (*d* = 0.86), for the entire therapy groups. However, the inter-therapy *d*-values were similar for the sub-samples of patients whose treatment was successful (*d* = 2.02–2.07) or unsuccessful (*d* = 0.39–0.73). The larger overall *d*-value for splint therapy reflects a value of success rate for splint therapy which, although not significant, was slightly larger than that for physiotherapy (Table 2). Thus slightly more patients with a successful treatment and therefore a larger *d*-value, contribute to the overall *d*-value of splint therapy.

### TDC outcome of effectiveness and success rate

Advantages of considering the post-treatment TDC outcome as a measure of effectiveness are an involvement of scores from various signs and symptoms of myogenous TMD rather than from solely pain intensity, and therefore also more statistical power to detect possible inter-therapy differences. The similar mean post-treatment TDC-values for physiotherapy and splint therapy (−0.512– −0.575, Table 3) indicate a similar effectiveness and a large effect size (TDC < −0.379) for both types of therapy. The post-treatment TDC-values were also similar between both therapies, in subgroups of patients for which treatment was successful or unsuccessful. The post-treatment TDC-values for patients whose treatment was unsuccessful (−0.200– −0.208) which were larger (less negative) than others, differed nevertheless significantly from zero (TDC = 0 means no change). Thus even in patients whose treatment was unsuccessful, their signs and symptoms improved significantly and similarly for both therapies. The success rate based on post-treatment TDC-values from individual patients is also similar between both therapies, *i.e.* 73–83% in the short-term and 51–60% in the long-term (Table 2).

### Considerations on Success Rate of Therapies

Patients with mainly basic signs and symptoms of myogenous TMD were selected in the present study. This selection is reflected in mean baseline values of various psycho-social factors which, in general, correspond to a low degree of involvement [23]. Significant differences did, in general, not occur between these baseline values for patients whose treatment became successful or unsuccessful respectively. Only internal locus of control differed for physiotherapy, *i.e.* patients whose physiotherapy became successful believed more that own health is controlled by their own behaviour than patients whose physiotherapy became unsuccessful. This outcome may be related to the degree of the patients’ active participation during physiotherapy. The difference in mean score level was however small, *i.e.* 9.2% with respect to the mutual mean. Baseline values of age or clinical variables from the present study did also not influence a patient’s responsiveness to a therapy. Regardless of the type of therapy, a modest use of OTC-medication (mean: 3–7% of the times of scoring) is not specifically related to any of the two treatment outcomes (successful/unsuccessful). Although larger patient groups are needed for more power in testing frequencies, this finding suggests that such a modest use may have only a small influence on success rate.

Despite a selection of ‘basic’ myogenous TMD patients, the success rates in the short-term (73–83%) were similar to that reported in text books for TMD in general [1, 38, 39]. The success rates in the long-term (51–60%) were such that a large fraction of the patients, on average 44%, still needed a subsequent treatment, regardless of the type of initial therapy. In contrast to previous studies, profoundly confounding factors which might hamper an improvement in central pain mechanisms, were excluded in the present study. A patient’s treatment may therefore become unsuccessful merely because the patient’s speed of recovery is intrinsically low. Post-treatment TDC-values indicate that even for patients whose treatment is unsuccessful, their TMD signs and symptoms have improved significantly. This improvement is also reflected as a significant decrease in pain intensity alone. The decrease in pain intensity does not continue beyond the end of treatment, as although not significant, the mean pain intensity was slightly larger at the first post-treatment visit than at the end of treatment about 4 weeks earlier (Fig. 2). For patients whose initial therapy is unsuccessful, stepped-care with a subsequent therapy is therefore necessary to attain a further decrease in pain intensity and other signs and symptoms beyond a critically low residual level.

### Preference of Initial Therapy in Stepped-Care

Our suggestions for an optimal treatment strategy refer to myogenous TMD which is not confounded by overt psychological problems, or severe sleep bruxism (SB) as diagnosed instrumentally [40]. A multi-therapy modality is more appropriate for patients with major psychological problems [20]. An occlusal appliance may still be preferred for active SB [40], because, apart from reducing SB, further extreme tooth wear is prevented.

In order to assess the outcome of stepped-care from the present study, a single telephone survey was carried out at the end of the project, 1–4 years following the end of a trajectory of 1–2 therapies [21]. This survey, using the anamnestic questionnaire with eight items from the TDC-procedure (assessing pain intensity by a 0–10 point score), revealed that 93% of all patients had no need for treatment anymore. This success rate for stepped-care corresponds with the mean of the range of overall success rates (91–95%) in the stepped-care model using the basic success rates of physiotherapy and splint therapy, and a value of the modulation factor *m* which is larger than one (*m = 1.5*; Table 5). This finding suggests that, regardless of the sequence of physiotherapy and splint therapy, the initial treatment triggers an improvement that, although insufficient for patients whose initial treatment becomes unsuccessful, enhances the success rate of the subsequent treatment.

Physiotherapy may be preferred as initial therapy over occlusal splint therapy in stepped-care of myogenous TMD, for four reasons. First, with a similar success rate and effectiveness, the duration of physiotherapy is, on average, 10.4 weeks shorter. Hence, patients whose initial physiotherapy is unsuccessful can continue earlier with subsequent treatment thus lowering the risk on a sustained chronicity of pain.

Second, the stepped-care model shows that small and even moderately large inter-therapy differences in basic success rate are not important. The overall success rate of stepped-care does not depend on the therapy sequence, irrespective of any inter-therapy difference in basic success rate, if the success rate of a subsequent therapy is not influenced by the preceding one (*m = 1*). For a wide range of *m*-values around *m = 1* (*0 < m < 2*), the difference in overall success rate between the two possible trajectories is smaller than the inter-therapy difference in basic success rate. Hence, the overall success rate of stepped-care hardly depends on therapy sequence. For basic myogenous TMD, an unsuccessful initial treatment most likely enhances the success rate of the subsequent treatment (*m ≈ 1.5*). The largest overall success rate of stepped-care is then even attained by choosing the therapy with the lower basic success rate as the initial therapy, hence physiotherapy.

Third, the same therapeutic techniques of physiotherapy can be applied to decrease pain or pain-related function impairment of other body parts than facial ones, like the neck and shoulders. Pain was not restricted to facial areas in 69% of the patients (Table 1). A broader application of physiotherapy might even increase its basic success rate. Fourth, at least in the Netherlands, the costs of a trajectory that starts with physiotherapy, are lower.

Physiotherapy might have a disadvantage, *i.e.* the number of visits is on average 7.1 larger than for splint therapy. If availability of a physiotherapist is limited, for example for patients living in rural areas, or if patients have limitations of transportation and/or time, splint therapy might still be preferred as initial treatment. However, technology-assisted interventions, including internet-based ones [41, 42], may diminish the disadvantage of physiotherapy. In-person treatment may, at least in part, be replaced by internet-assisted treatment.

### Limitations

This study includes some limitations. First, patients with mainly basic signs and symptoms of solely myogenous TMD were selected to facilitate straightforward conclusions on therapy outcome. The prevalence of solely myogenous TMD is low in samples of patients who are referred to a specialized clinic, for example, 4.5% of all TMD patients in a tertiary clinic [43], and 9.0% in the present study where patients were mainly (85%) referred to a clinic for Special Dental Care. However, this prevalence is much higher in a more general clinic, *i.e.* 39.5% in an outpatient physical therapy practice (myogenous TMD without arthralgia) [44], and 47.8% in a university dental clinic [45]. The prevalence is also higher, 36.1%, in a community sample of young women (age 19–23 years) [46]. Hence, the results of the present study are representative for about 40–48% of all TMD patients, *i.e.* patients who have an age range of 18–65 years (median at about 32 years), and a need of treatment for solely myogenous TMD. This conclusion is reinforced by the finding that the patients from the present study are largely comparable to those with myogenous TMD from previous studies in which selection was less amended (for a detailed discussion, see ref [23]).

Second, patient groups with placebo treatments or a long waiting period were not included for ethical reasons. Using a placebo group in non-pharmacological studies may be theoretically, methodologically, practically, and ethically unsound [47]. Regarding a placebo effect of occlusal splint therapy, the effectiveness of a splint which does not cover the teeth but only the palate (palatal splint) is either similar [12], or corresponds in part to the effectiveness of an occlusal splint [15, 33]. However, occlusal splint therapy and physiotherapy have in common an extensive non-noxious mechanical stimulation of peri-oral and/or intra-oral tissues. A palatal splint also causes mechanical stimulation of intra-oral tissues. Non-noxious mechanical stimulation, hence stimulation of afferent A-β fibres, may relieve pain by modulation of central pain mechanisms [48, 49]. In order to avoid any stimulation of tissues, a sham laser treatment may be an appropriate placebo treatment. Recording the patients’ expectation on improvement will then be useful for assessing the role of this factor in effectiveness of various treatment modalities. Recording this expectation will also solve the third limitation of the present study.

Although a non-treatment group was not included in the present study, two waiting periods of about 4 weeks were present in both treatment modalities, before and after treatment. These waiting periods were long enough to show, in addition with a 2-week-pain diary, that the effect of one-time counselling at the initial visit (all patients) stabilized as well as the effect of an unsuccessful treatment. An initial treatment was stepped up for ethical reasons, when it was unsuccessful at a post-treatment occasion of evaluation. Because the decrease of pain intensity was halted following the end of an unsuccessful initial treatment, it is unlikely that the success rate and the last TDC-value would have altered by a spontaneous improvement if a follow-up of 1 year were completed. In a study using a waiting list of 1 year [34], intensity of various types of pain did not change in myogenous TMD patients.

Bias by regression to the mean is avoided by using TDC-values as outcome parameter [19]. TDC is therefore useful in future research to examine changes of all pronounced signs and symptoms during all phases, thus from the initial visit up to the end of a possible second treatment of a trajectory.

## Conclusions

Physiotherapy and occlusal splint therapy have similar success rates and effectiveness. Because the duration of physiotherapy is on average 10.4 weeks shorter than that of splint therapy, physiotherapy may be preferred as initial therapy over occlusal splint therapy in stepped-care of basic myogenous TMD which is not confounded by major psychological problems or severe active sleep bruxism. Patients whose initial physiotherapy is unsuccessful can then continue earlier with subsequent treatment. The stepped-care model reinforces the conclusion on therapy preference as the overall success rate of stepped-care in which possibly two successive therapies are involved, hardly depends on therapy sequence.

## Additional files

Additional file 1: Appendix. Threshold of signs and symptoms. Post-hoc power analysis on measures of effectiveness. Two-way ANOVA statistical analysis for pain intensity. Rules for progressing and ending splint therapy. Rules for progressing and ending physiotherapy. A stepped-care model including two possible therapies. (PDF 86 kb)
Additional file 2: Dataset. Dataset related to Tables 1-4. (XLSX 1434 kb)

## Abbreviations

ANOVA: Analysis of variance
CD: Clinical difference
CID: Clinically important difference
*d*: Cohen’s difference (measure of effect size)
EM: End measurement of treatment outcome at the first post-treatment visit
E-Tx: End of treatment
I: Initial visit
LM: Last measurement of treatment outcome at the last post-treatment visit
*m*: Modulation factor in the stepped-care model
NS: Non-significant
OTC medication: Over-the-counter medication
Ph-Tx: Physiotherapy
RCT: Randomized controlled trial
RDC/TMD: Research diagnostic criteria for temporomandibular disorders
SB: Sleep bruxism
SD: Standard deviation
SDD: Smallest detectable difference
Sp-Tx: Occlusal splint therapy
SR: Success rate
SR_tr_: Success rate of a trajectory of 1–2 therapies in the stepped-care model
St-Tx: Start of treatment
STx: Successful treatment
TDC: Treatment duration control
TMD: Temporomandibular disorders
Tx: Treatment
UTx: Unsuccessful treatment
VAS: Visual analogue scale
