# Supplementary material for: Towards an optimal therapy strategy for myogenous TMD, physiotherapy compared with occlusal splint therapy in an RCT with therapy-and-patient-specific treatment durations
Source: BMC Musculoskelet Disord. 2017 Feb 10;18:76. doi: 10.1186/s12891-017-1404-9 (PMC5301345; doi:10.1186/s12891-017-1404-9)
Supplement: Additional file 1: — Appendix. Threshold of signs and symptoms. Post-hoc power analysis on measures of effectiveness. Two-way ANOVA statistical analysis for pain intensity. Rules for progressing and ending splint therapy. Rules for progressing and ending physiotherapy. A stepped-care model including two possible therapies. (PDF 86 kb) [file 12891_2017_1404_MOESM1_ESM.pdf]

Towards an optimal therapy strategy for myogenous TMD, physiotherapy compared with occlusal splint therapy in an RCT with therapy-and-patient-specific treatment durations

*Robert J van Grootel, Rob Buchner, Daniël Wismeijer, Hilbert W van der Glas**

*Correspondence: h.vanderglas@dundee.ac.uk; hwvanderglas@hotmail.com

Additional file 1: Appendix

Threshold of signs and symptoms

Regarding a threshold of signs and symptoms at the initial visit and the start of treatment, a patient had to meet criteria in two areas of clinical examination. First, when examining active and passive jaw movements, included patients had at least one score of '3' of pain intensity ('severe pain') or at least three scores of '2' ('moderate pain'). Second, when carrying out palpation of jaw muscles or for the anamnestic questions, the intake threshold included at least two scores of '3' related to intensity or frequency of pain or impairment ('severe pain', 'often painful', or 'often impairment'). For muscle palpation, more than one score of '3' had to be related to remotely located jaw muscle 'units'. For example, the deep and superficial masseter muscles on the right-hand side were considered as one muscle unit regarding number of threshold scores of muscle palpation, and the deep masseter muscle on the right-hand side and the superficial masseter muscle on the left-hand-side as two units.

Post-hoc power analysis on measures of effectiveness

The present study uses two measures of therapy effectiveness, *i.e.* the mean last post-treatment TDC-value and the difference in mean pain intensity, between the last post-treatment measurement and that at the start of treatment. Because inter-therapy differences were non-significant and TDC is a novel variable, it is meaningful to analyse the post-hoc power of the present study for both measures.

Considering the number of 35-37 included patients, a mean TDC-value of -0.575 for the entire group of splint therapy (reference value, *cf.* Results, Table 3) and a common SD value of 0.350 for physiotherapy and splint therapy, this study has post-hoc 68% power at a level of 5% type I errors to detect an inter-therapy difference of 35.0% in the mean TDC-value, and 80% power to detect a difference of 40.0%.

Enhancements of 35.0% and 40.0% in mean TDC-values (TDC less negative, a second therapy is then less effective than splint therapy) are equivalent to a reduction of 25.5% and 29.7% respectively of the difference between scores from a single variable, like ‘pain intensity’, between the start of treatment and the last post-treatment measurement (see below). Reductions of 35% and 40.0% in mean TDC-values (TDC more negative, a more effective second therapy) are equivalent to an enhancement of 19.7% and 22.2% of the difference in scores of ‘pain intensity’, before and after treatment. Hence, the power of the present study was sufficiently large for detecting fairly small inter-therapy differences in effectiveness.

As an example of the calculation of the equivalence between an enhancement in TDC and a reduction in the difference in values of ‘pain intensity’: the mean TDC-value of splint therapy (reference of effectiveness) is -0.575 (*cf.* Results, Table 3). A TDC-value of -0.575, a mean of contrast values from several items [19], is equivalent to a single Contrast value, *C*, of -0.575 from two normalized values of ‘pain intensity’, *i.e.* 100 at the start of treatment (100%) and 27 at the last post-treatment measurement (C = (27-100)/27+100)= -0.575). The difference between the first and the second value of ‘pain intensity’ is then 73 (100-27). An enhancement of 35% of the TDC-value of -0.575 yields a TDC-value of -0.374, which is equivalent to the Contrast value of the values 100 and 45.6 of ‘pain intensity’ (C = (45.6-100)/(45.6+100) = -0.374). The difference between both values of ‘pain intensity’ is then 54.4. Hence, enhancing the TDC-value by 35% is equivalent to a reduction by 25.5% of the difference between pre-treatment and post-treatment values of ‘pain intensity’ (=54.4-73)/73x100 %). The amount of percentage change differs between TDC and the difference between two single scores of a variable because contrast values equal approximately logarithmic values of ratios between scores [19], while a difference is related to scores of which the numerical values are ranked on a linear scale.

For a post-hoc power assessment of a detectable treatment effect on the difference from single scores of pain intensity which were actually obtained (*cf.* Results, Table 4), individual difference values were considered between the last post-treatment measurement and the start of treatment. Considering the number of 35-37 included patients, a mean difference value of 27.7 scale-% for the entire group of splint therapy and a common SD value of 24.4 for physiotherapy and splint therapy, this study has 39% power at a level of 5% type I errors to detect an inter-therapy difference of 35.0% in the mean difference value of pain intensity, and 80% power to detect a difference of 58.4%.

Considering the exchange rates between changes in TDC-values and changes in difference values from ‘pain intensity’ before and after treatment (see above), the power is larger for TDC-

values (a multidimensional variable) than for differences in the actual scores of pain intensity (a unidimensional variable).

Two-way ANOVA statistical analysis for pain intensity

Table S6 Statistical testing of levels of predominant pain intensity from the masticatory system, presented in Table 4 for different therapies and stages

two-way ANOVA		
therapy (p-value/level)	stage (p-level)	interaction (p-value/level)
0.1562 NS	<0.0001	0.5328 NS

Bonferroni's multiple comparison tests on stage-differences per therapy group:

	physiotherapy (p-level)	splint therapy (p-value/level)
initial visit vs start-Tx	<0.0001	0.0013 <0.01
start-Tx vs LM	<0.0001	<0.0001
initial visit vs LM	<0.0001	<0.0001

Two-way ANOVA (p-values/significance levels, NS, non-significant) with the factor therapy (unpaired observations, 2 levels), and procedure stage (paired observations, 3 levels). Stages: the initial visit, the start of treatment (start-Tx) and the last post-treatment measurement of pain intensity (LM).

Table S7 Statistical testing of levels of predominant pain intensity depicted in Figure 2 for 2 therapies, 2 treatment outcomes and 4 stages

physiotherapy (n=37) two-way ANOVA (p-value/level)			splint therapy (n=35) two-way ANOVA (p-value/level)		
Tx-outcome u.o.	stage p.o.	interaction	Tx-outcome u.o.	stage p.o.	interaction
0.0267 <0.05	<0.0001	0.0032 <0.01	0.0695 NS	<0.0001	0.0025 <0.01

Bonferroni's multiple comparison tests on differences between Tx-outcome groups per stage (u.o.):

		physiotherapy (p-value/level)		splint therapy (p-value/level)	
I:	STx vs UTx	>0.9999	NS	>0.9999	NS
St-Tx:	STx vs UTx	0.5705	NS	>0.9999	NS
E-Tx:	STx vs UTx	0.0232	<0.05	0.0907	NS
EM:	STx vs UTx	0.0063	<0.01	0.0031	<0.01

Bonferroni's multiple comparison tests on differences between stages (p.o.) for STx and UTx:

	physiotherapy (p-value/level)		splint therapy (p-value/level)	
STx: I vs St-Tx		<0.0001	0.005	<0.01
STx: St-Tx vs E-Tx		<0.0001		<0.0001
STx: St-Tx vs EM		<0.0001		<0.0001
STx: E-Tx vs EM	>0.9999	NS	>0.9999	NS
UTx: I vs St-Tx	0.1339	NS	0.025	<0.05
UTx: St-Tx vs E-Tx	0.0016	<0.01	0.0004	<0.001
UTx: St-Tx vs EM	0.0018	<0.01	0.0077	<0.01
UTx: E-Tx vs EM	>0.9999	NS	>0.9999	NS

STx (n=40) two-way ANOVA (p-value/level)			UTx (n=32) two-way ANOVA (p-value/level)		
therapy u.o.	stage p.o.	interaction	therapy u.o.	stage p.o.	interaction
0.4660 NS	<0.0001	0.2708 NS	0.3061 NS	<0.0001	0.9531 NS

Treatment (Tx) outcome: successful treatment (STx) and unsuccessful treatment (UTx) according to TDC. Stages: initial visit (I), start of treatment (St-Tx), end of treatment (E-Tx), and end measurement of treatment at the first post-treatment visit (EM). Number of patients in the four subgroups: n=19 for physio STx, n=21 for splint STx, n=18 for physio UTx and n=14 for splint UTx.
Top: 2-way ANOVAs (p-values/significance levels; NS, non-significant) for physiotherapy and splint therapy respectively, with 'Tx-outcome' (2 levels) and 'stage' (4 levels) as factors. u.o. and p.o.: unpaired observations and paired observations respectively.
Bottom: 2-way ANOVAs (p-values/levels) for successful treatment (STx) and unsuccessful treatment (UTx) respectively, with 'therapy' (2 levels) and 'stage' (4 levels) as factors. Bonferroni's multiple comparison tests on differences between stages (p.o.) for STx and UTx showed a similar pattern of significance as shown in the top part of the Table for the other 2-way ANOVAs.

Rules for progressing and ending splint therapy

1) The intended visit program includes maximally 5 visits (including the start visit, week 0) with intervals of 6 weeks and minimally 2 visits. The intended maximal duration of wearing a splint each night is thus 24 weeks while the intended minimal duration of wearing is 6 weeks.
   TDC is determined by the clinician at each possible visit, first to decide when the splint can gradually been withdrawn (paragraph 2), and second to regard the progress of treatment.
   If TDC is > -0.212 following the first 3 visits, treatment with a splint is ended because the patient is not sufficiently responsive (paragraph 3).
   If following 3 visits or more, TDC is ≤ -0.379 (reaching functional status) at two successive visits and the discrepancy rule[†] does not apply, treatment is ended as being potentially successful (paragraph 4).
   If -0.379 < TDC ≤ -0.212, a patient is sufficiently responsive, and treatment is continued if the preset maximal number of 5 visits is not exceeded. If the maximal number of 5 visits is reached, treatment is finished (paragraph 5). Patients whose treatment is ultimately considered by the clinician as being potentially successful or unsuccessful, are transferred to the assessor for blinded evaluation and the decision regarding treatment success for the randomized controlled trial;
2) Withdrawal of wearing the splint can start at the 2nd visit (week 6)[§] or at a later visit if TDC ≤ -0.379 and the discrepancy rule[†] does not apply. This withdrawal has a total duration of 6 weeks and is gradually carried out (week 1, 1 night less wearing; week 2, 2 nights less etc.). If the patient's status appears to have worsened above the upper limit of functional status (TDC>-0.379) following 6 weeks of withdrawal, full wearing is resumed for the next 6 weeks;
3) If at the 3rd visit or at a later one (week 12 or later) the patient's responsiveness to treatment is insufficient (TDC > -0.212), the patient is asked for compliance of splint wearing. If compliance is sufficient or cannot be fulfilled, splint treatment is then ended as potentially being unsuccessful;
4) At the 3rd visit or at a later one (week 12 or later), patients might have a functional status at this visit and the previous one (*i.e.* TDC≤ -0.379 and no application of the discrepancy rule[†] for a period of 12 weeks). Furthermore withdrawal of the splint will have been completed at this stage (paragraph 2). Treatment of these patients is then ended as being potential successful;

5) At the 5th visit (week 24), treatment is ended for the remaining patients. The clinician determines the patient's status and decides as follows:

*If the patient has functional status (TDC≤ -0.379 and no application of the discrepancy rule[†]) while withdrawal of the splint (paragraph 2) is completed, treatment is considered as being potentially successful;

*If the patient has functional status while the patient is still wearing the splint, the withdrawal process is started and controlled 6 weeks later (additional 6th visit of the program, at week 30). If the patient has still functional status, the treatment is considered as being potentially successful. If the patient's status has worsened following withdrawal, treatment is considered as being potentially unsuccessful;

*If the patient has not attained functional status (TDC > -0.379), treatment is considered as being potentially unsuccessful.

[§]Each visit is related to a week number with respect to the start of treatment (1st visit of treatment, week 0).

[†] The following option has been added to the procedures of TDC-outcome to comply with usual clinical care and for ethical reasons. The patient's opinion as reflected in anamnestic items on daily functioning of the oral system was given priority in the treatment outcome if the index of overall relative change (including changes related to items from clinical tests) indicated a 'successful' treatment while the anamnestic items alone indicated an 'unsuccessful' treatment. To that end, the following 'discrepancy rule' was applied to the decisions of the clinician as well as conclusions of the investigator based on the data from the assessor. If the overall TDC was ≤ -0.379 (successful treatment), but TDC-anamnestic-items was >-0.212 (treatment with insufficient effect according to the patient), the treatment was considered as unfinished or, if further continuation of the treatment was not possible, the treatment was considered as being unsuccessful. The discrepancy rule was only occasionally applied, *i.e.* in 2.9 % of the patients (1 out of 35) with splint therapy.

Rules for progressing and ending physiotherapy

1) The intended visit program includes maximally 15 visits (including the start visit, week 0) with intervals of 0.5 - 6 weeks and minimally 10 visits. The maximal duration of physiotherapy is 21 weeks while the minimal duration is 10 weeks. Patients whose treatment is ultimately considered by the clinicians (physiotherapist and responsible dentist) as being potentially successful or unsuccessful, are transferred to the assessor

for blinded evaluation and the decision regarding treatment success for the randomized controlled trial;

2) The maximal treatment program includes stage (i) a start visit (week 0) with only intake for physiotherapy, and stage (ii), 6 visits with a frequency of 2 visits/week, thus 3 weeks in total, during which exercises are instructed to be carried out at home and their performance is controlled during the visits. At the end of stage (ii) (week 3) the physiotherapist determines TDC. For patients of the maximal program, treatment will not be successful ($-0.379 < \text{TDC} \leq -0.212$ or $\text{TDC} \leq -0.379$ but the discrepancy rule[†] does apply). Treatment is then continued with stage (iii), 6 visits with a frequency of 1 visit/week during which the performance is controlled of specific exercises which have been carried out at home (end of this stage at week 9). Furthermore the physiotherapist determines TDC at each visit of stage (iii) with the conclusion of a non-successful treatment for patients of the maximal program. Stage (iii) is followed by stage (iv), 2 visits within a week in which the patient's status is subsequently determined by the clinician (the physiotherapist) and the dentist who is responsible for the patient (week 9-10). With a non-successful treatment at stage (iv), either according to the physiotherapist and/or the responsible dentist, stage (iv) is followed by stage (v), a final period of 6 weeks during which specific exercises are carried out at home. Stage (v) is followed by stage (vi), two visits within a week for determining the patient's status by the physiotherapist and the dentist respectively (end at week 21). Patients from the maximal program might then have a potentially successful treatment ($\text{TDC} \leq -0.379$ and the discrepancy rule[†] does not apply) according to the physiotherapist as well as the dentist. However, regardless of treatment outcome from the clinicians at stage (vi), the patients are then referred to the blinded assessor;

3) The minimal program includes the components under (i), (ii) and (iv), with specific exercises carried out at home between stage (ii) and (iv), however without intermediate visits for control. TDC is determined by the clinician (physiotherapist) at the start visit, at the end of stage (ii) (week 3), and 6 weeks later at stage (iv) (week 9 for the minimal program rather than week 15 for the maximal program). For the minimal program, the outcome of the TDC-procedure will always be $\text{TDC} \leq -0.379$ and the discrepancy rule[†] does not apply. At stage (iv), TDC is also determined by the dentist who is responsible for the patient.

4) The intermediate program differs from the maximal program in that stage (iii) can be shortened and/or stage (v) can be absent. Patients whose treatment is not successful at

the end of stage (ii) (week 3; -0.379 < TDC ≤ -0.212 or TDC ≤ -0.379 but the discrepancy rule† does apply), have a TDC-assessment every week of stage (iii), as long as treatment remains unsuccessful. Thus possible moments of TDC- assessment occur at week 4, 5, 6, 7, 8 and 9. Once treatment has become successful, specific exercises are carried out at home for six weeks without further intermediate visits for TDC-assessment, reaching stage (iv) at week 10-15. Stage (iv) includes 2 visits within a week in which the patient's status is subsequently determined by the clinician (the physiotherapist) and the dentist who is responsible for the patient (end week 10-15). If treatment is potentially successful according to the physiotherapist as well as the dentist (TDC ≤ -0.379 and the discrepancy rule† does not apply), then the patient is referred to the assessor. Otherwise, the patient continues with stage (v) a final period of 6 weeks during which specified exercises are carried out at home. Stage (v) is then followed by stage (vi), two visits within a week for determining the patient's status by the physiotherapist and the dentist respectively (end at week 16-21). The patients are then referred to the assessor.

---

†see the endnote of Appendix, 'Rules for progressing and ending splint therapy', for further explanation of the discrepancy rule. The discrepancy rule was only occasionally applied, *i.e.* in 2.7 % of the patients (1 out of 37) with physiotherapy.

A stepped-care model including two possible therapies

Suppose that a trajectory of stepped-care consists of a first type of therapy '*A*', which if unsuccessful, is followed by a second type of therapy '*B*'. The number of patients for which therapy *A* is successful ($n_{A,S}$) is given by:

$n_{A,S} = n_T.f_{A,S}$ (equation (1))

in which n_T is the total number of patients in the trajectory and $f_{A,S}$ is the fraction of patients for which therapy *A* is successful (success rate as a fraction = percentage SR/100).The number of patients for which therapy *A* is unsuccessful ($n_{A,U}$) is then given by:

$n_{A,U} = n_T.(1\text{-}f_{A,S})$ (equation (2))

Therapy *A* is followed by therapy *B* for these patients and the number of patients for which this subsequent therapy *B* is successful ($n_{B,S}$) is given by:

$n_{B,S} = n_{A,U}.f_{B,S}.m$ (equation (3))

in which $f_{B,S}$ is the fraction of patients for which therapy *B* is successful if this therapy were applied separately (thus without a preceding therapy *A*, yielding the 'basic' fractional success rate), and *m* is a modulation factor which describes the possible influence of a preceding therapy *A* on the basic success rate of therapy *B* ($m \geq 0$).

A value of $m=0$ means that all patients whose treatment was unsuccessful following the first therapy *A* will also have an unsuccessful treatment with the subsequent therapy *B*. A value of $m=1$ means that an unsuccessful preceding therapy will not influence the basic success rate of the subsequent therapy. Values of *m* between 0 and 1 ($0 < m < 1$) indicate a diminished success rate of the subsequent therapy. This diminishing may occur if following unsuccessful treatments, patients remain who are less or more slowly responsive to any subsequent therapy. The success rate of a subsequent therapy may be enhanced, thus $m > 1$, if the initial therapy *A* starts a process of improvement which is continued and enhanced by a subsequent therapy *B*. When this improvement is continued in a slowly responsive patient, functional status may be attained, merely by a longer treatment duration following two subsequent therapies. Improvement by therapy *B* might further be enhanced, for example, if the patient's expectation of improvement would be increased by using a new therapy. The modulation factor *m* in the model reflects the net effect of various factors.

From equations (1) and (3) it follows that the number of patients for whom therapy *A* alone or therapy *B* (following an unsuccessful therapy *A*) is successful ($n_{A,S} + n_{B,S}$) is given by:

$n_{A,S} + n_{B,S} = n_T.f_{A,S} + n_{A,U}.f_{B,S}.m$ (equation (4))

Substitution of equation (2) in equation (4) yields:

$n_{A,S} + n_{B,S} = n_T.f_{A,S} + [n_T.(1\text{-}f_{A,S})].f_{B,S}.m$

Thus the overall fractional success rate of the entire therapy trajectory which consists of therapy *A* possibly followed by therapy *B* is given by:

$(n_{A,S} + n_{B,S})/n_T = f_{A,S} + m.f_{B,S} – m.f_{A,S}.f_{B,S}$ (equation (5)).

The success rate (SR_{tr}) expressed as a percentage of the patients whose trajectory is successful (= $[(n_{A,S} + n_{B,S})/n_T).100]$ %) is given by:

$SR_{tr} = (f_{A,S} + m.f_{B,S} – m.f_{A,S}.f_{B,S}).100$ % (equation (6))

Because the maximal possible value of the success rate of a trajectory is 100%, the value of *m* is also bound to a maximum. Substituting the value of 1 for the maximal fractional success rate of the trajectory in equation (5) yields:

$1 = f_{A,S} + m.f_{B,S} – m.f_{A,S}.f_{B,S}$, from which it follows that:

$m_{max}=1/f_{B,S}$

Thus the maximal value of *m* equals the inversed value of the fractional basic success rate of the second therapy in the trajectory.

If $m=1$, equation (5) reduces to:

$(n_{A,S} + n_{B,S})/n_T = f_{A,S} + f_{B,S} - f_{A,S}.f_{B,S}$ (equation (7)),

and the success rate (SR_{tr}) expressed as a percentage of the patients whose trajectory is successful (= $[(n_{A,S} + n_{B,S})/n_T).100]$ %) is then given by:

$SR_{tr} = (f_{A,S} + f_{B,S} - .f_{A,S}.f_{B,S}).100$ % (equation (8))

The terms $f_{A,S}$ and $f_{B,S}$ can then be interchanged in the sum part as well as in the product part of equation (7) or equation (8) without influencing the outcome for the overall success-rate of the entire therapy trajectory. Hence, with a reversed sequence of possible therapies in the alternative trajectory, *i.e.* starting with therapy *B* followed by therapy *A* when therapy *B* is unsuccessful, equation (7) can then be rewritten as:

$(n_{B,S} + n_{A,S})/n_T = f_{B,S} + f_{A,S} - f_{B,S}.f_{A,S}$, without changing the outcome.

Thus from equation (7) or equation (8) it follows that in the absence of a net effect of a preceding therapy on the success rate of a subsequent therapy ($m=1$), the success rate of the entire trajectory will not depend on the sequence in which the two types of therapies are applied. This invariance of sequence of application will occur regardless of a possible difference in the basic success rate between the two therapies. As an example, suppose that the success rate is 70% for therapy *A* and 50% for therapy *B*. The overall success rate of a trajectory that starts with therapy *A* followed by therapy *B* when therapy *A* is unsuccessful, is then 85% according to equation (8). This overall success rate will also occur with a reversed sequence of possible therapies in the alternative trajectory, *i.e.* starting with therapy *B* followed by therapy *A* when therapy *B* is unsuccessful.

If the success rate of a subsequent therapy is decreased following the application of an unsuccessful preceding therapy ($m<1$), the overall success rate of a trajectory will depend on the sequence of application of the two therapies. The largest overall success rate will then occur in that trajectory in which the starting therapy has the largest basic success rate (equation (5)). Under the assumption of identical *m*-values regardless of therapy sequence in a trajectory (which is plausible for therapies of which the success rates, although not identical, are similar when applied separately), the overall fractional success rate in a trajectory with reversed therapy sequence is given by:

$(n_{B,S} + n_{A,S})/n_T = f_{B,S} + m.f_{A,S} - m.f_{B,S}.f_{A,S}$ (equation (9))

The difference in overall success rate between two trajectories with reciprocal therapy sequences follows from subtraction of equation (5) with equation (9); this difference, denoted by Δ, is given in an absolute sense by:

$\Delta = \left| (1-m).(f_{A,S}-f_{B,S}) \right|$ (equation (10))

in which $(f_{A,S}-f_{B,S})$ corresponds with the difference in basic fractional success rate when both types of therapies are applied separately. When $(f_{A,S}-f_{B,S})$ is denoted as Δ_0, equation (10) can be rewritten as:

$\Delta = \left| (1-m).\Delta_0 \right|$ (equation (11))

Equation (11) shows that for the range of *m*-values given by $0<m\leq 1$, the difference in overall success rate between trajectories with reciprocal therapy sequences (Δ) is smaller than the difference between the success rates of the therapies when applied separately (Δ_0).

As an example, with a basic fractional success rate of 0.70 for therapy *A* and 0.50 for therapy *B*, the difference in success rate is 0.20 (20%) when both therapies are applied separately. Suppose that only half of the basic success rate of a therapy is realized when this therapy occurs as the second one in a trajectory of two therapies, thus the value of the modulation factor *m* equals 0.50. The difference in overall success rate between the two trajectories with reciprocal therapy sequences (0.10; 10%; equation (11)) will then be halved with respect to the difference in separate success rate (0.20; 20%).

If the success rate of a subsequent therapy is increased ($m>1$), the difference Δ in overall fractional success rate between two trajectories with reciprocal therapy sequences is also given by equation (11). From equation (11) it follows that for the range of *m*-values given by $1<m\leq 2$, the Δ-value is, like for the range $0<m<1$, also smaller than the difference in basic success rates, Δ_0. In contrast to the range of $0<m<1$, the largest overall success rate of the trajectory will occur for $1<m\leq 2$ when this trajectory is started with the therapy which has the smallest rather than the largest basic success rate.
